# Complete Genome Sequence of *Streptococcus agalactiae* CNCTC 10/84, a Hypervirulent Sequence Type 26 Strain

**DOI:** 10.1128/genomeA.01338-14

**Published:** 2014-12-24

**Authors:** Thomas A. Hooven, Tara M. Randis, Sean C. Daugherty, Apurva Narechania, Paul J. Planet, Hervé Tettelin, Adam J. Ratner

**Affiliations:** a Department of Pediatrics, Columbia University, New York, New York, USA; b Institute for Genome Sciences, University of Maryland School of Medicine, Baltimore, Maryland, USA; c Sackler Institute for Comparative Genomics, American Museum of Natural History, New York, New York, USA

## Abstract

*Streptococcus agalactiae* (group B *Streptococcus* [GBS]) is a human pathogen with a propensity to cause neonatal infections. We report the complete genome sequence of GBS strain CNCTC 10/84, a hypervirulent clinical isolate frequently used to study GBS pathogenesis. Comparative analysis of this sequence may shed light on novel pathogenic mechanisms.

## GENOME ANNOUNCEMENT

*Streptococcus agalactiae* (group B *Streptococcus* [GBS]) is a leading cause of neonatal sepsis and an emerging pathogen in adults (1, 2). Complete genome sequences of several GBS strains have been reported, and comparative genomic analyses have been extremely valuable in understanding GBS evolution (3). The strain CNCTC 10/84 was originally isolated from human clinical samples by Wilkinson and designated 1169-NT1 (4). It has been characterized as capsular polysaccharide type V (5) and sequence type 26 (ST-26) (6). Because of its highly virulent phenotype in animal models and its overproduction of β-hemolysin/cytolysin (βHC), CNCTC 10/84 has been widely used in studies of GBS-host interactions over the past two decades (7–10).

Genomic DNA from *S. agalactiae* CNCTC 10/84 (ATCC 49447; CCUG 29784) was sequenced using Illumina MiSeq sequencing (paired end 2 × 150 bp; 3,726,676 total reads; 142.5-bp average length; estimated genome coverage 260×). Reads were assembled using Celera Assembler version 7.0 (11), resulting in 9 scaffolds with a cumulative size of 1,988,721 bp. The remaining gaps were closed using PCR and Sanger sequencing. Annotation was performed using the IGS Prokaryotic Annotation Engine (12). The final GBS CNCTC 10/84 genome sequence is 2,013,842 bp, has a GC content of 35.4%, 80 tRNA genes, 7 rRNA operons, and 1,980 predicted coding sequences.

We confirmed the ST-26 classification of CNCTC 10/84 using the PubMLST server (http://pubmlst.org/sagalactiae) (13). Consistent with prior reports, the CNCTC 10/84 genome sequence contains a capsular polysaccharide synthesis locus most similar to those of other type V strains. Prior studies suggested that CNCTC 10/84 is phenotypically similar to GBS strains deficient in the *covRS* (*csrRS*) two-component system (6). However, we noted the presence of an intact *covRS* locus as well as additional two-component systems. The GBS *cyl* locus (*cylX*-*cylK*) is responsible for the synthesis of the granadaene pigment and βHC (14, 15), which may be the same molecule (16). We found an intact *cyl* locus and promoter region in the CNCTC 10/84 genome. Other factors important in GBS-host interactions were present in the CNCTC 10/84 genome, including pilus biosynthesis machinery, a eukaryotic-like serine–threonine kinase/phosphatase pair, sortases, adhesins, and other predicted cell wall–anchored proteins. Comparative analysis of the GBS CNCTC 10/84 genome with other available GBS genomes may inspire testable hypotheses regarding the basis for this strain’s hypervirulence.

### Nucleotide sequence accession number.

This whole-genome sequence and annotation are available at GenBank under the accession number CP006910.
